# Optical Coherence Tomography Imaging in Acute Coronary Syndromes

**DOI:** 10.4061/2011/312978

**Published:** 2011-09-21

**Authors:** Takashi Kubo, Yasushi Ino, Takashi Tanimoto, Hironori Kitabata, Atsushi Tanaka, Takashi Akasaka

**Affiliations:** Department of Cardiovascular Medicine, Wakayama Medical University, 811-1 Kimiidera, Wakayama 641-8510, Japan

## Abstract

Optical coherence tomography (OCT) is a high-resolution imaging technique that offers microscopic visualization of coronary plaques. The clear and detailed images of OCT generate an intense interest in adopting this technique for both clinical and research purposes. Recent studies have shown that OCT is useful for the assessment of coronary atherosclerotic plaques, in particular the assessment of plaque rupture, erosion, and intracoronary thrombus in patients with acute coronary syndrome. In addition, OCT may enable identifying thin-cap fibroatheroma, the proliferation of vasa vasorum, and the distribution of macrophages surrounding vulnerable plaques. With its ability to view atherosclerotic lesions in vivo with such high resolution, OCT provides cardiologists with the tool they need to better understand the thrombosis-prone vulnerable plaques and acute coronary syndromes. This paper reviews the possibility of OCT for identification of vulnerable plaques in vivo.

## 1. Introduction

**Figure 1:**
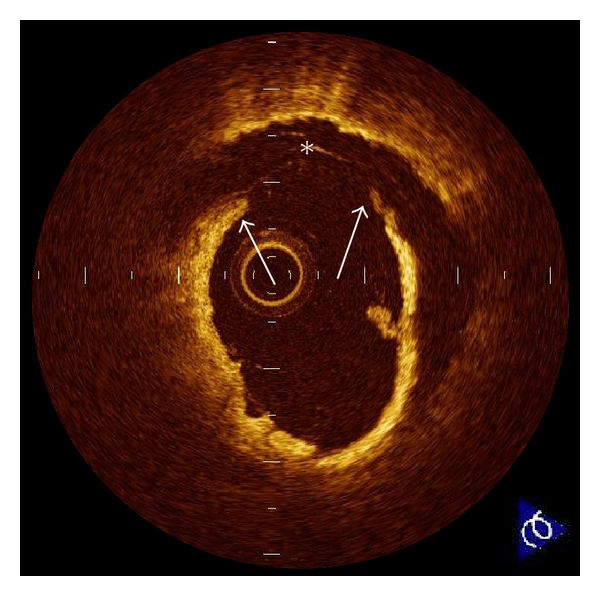
Plaque rupture. Plaque rupture is defined as a presence of fibrous-cap discontinuity (arrows) and a cavity formation (∗) in the plaque. Ruptured plaques usually have an extensive lipid core and a thin fibrous cap. The fibrous cap is the thinnest at the site of rupture, and the plaque cavity indicates loss of lipid core due to rupture.

**Figure 2:**
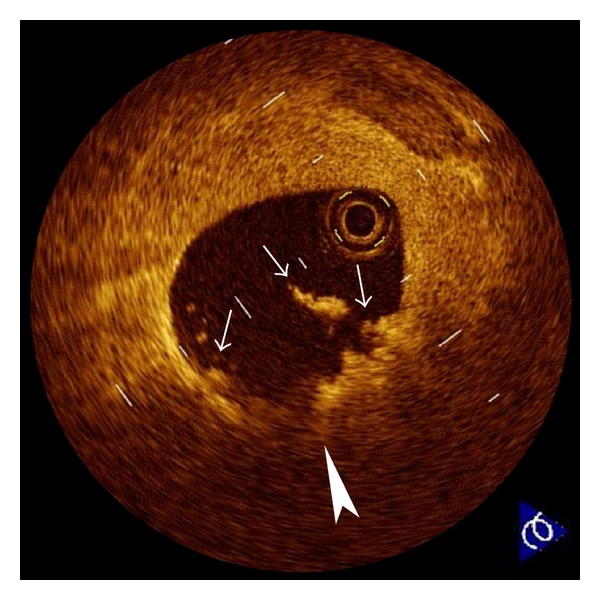
Plaque erosion. Erosion (arrowhead) is usually comprised of OCT evidence of thrombi (arrows), an irregular luminal surface, and no evidence of cap rupture evaluated in multiple adjacent frames. The characteristic of OCT features of erosions are a thick intima and a small or absent lipid core. If the lipid core is present, it does not communicate with luminal thrombi.

**Figure 3:**
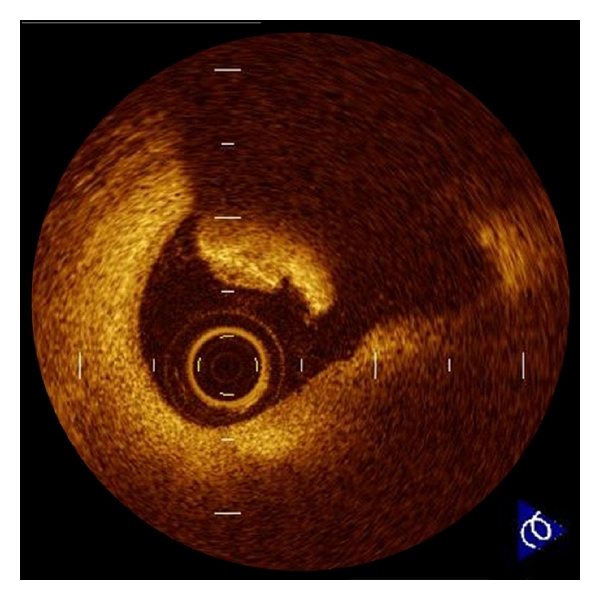
Intracoronary thrombus. Thrombus is defined as a protrusion inside the lumen of the artery with signal attenuation. White thrombus which consists mainly of platelets is identified as signal rich, low-backscattering protrusions in the OCT image, while red thrombus which consists mainly of red blood cells is identified as high-backscattering protrusions inside the lumen of the artery, with signal-free shadowing in the OCT image.

**Figure 4:**
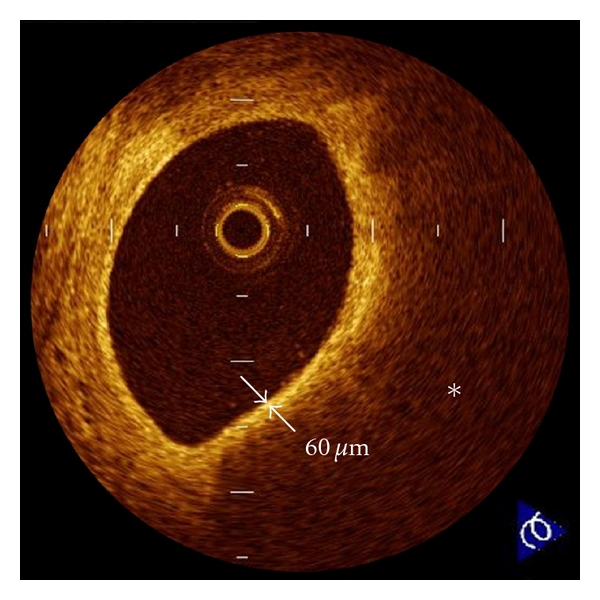
Thin-cap fibroatheroma (TCFA). A fibrous cap (arrows) is identified as a signal-rich homogenous region overlying a lipid core (∗), which is characterized by a signal-poor region. TCFA is defined as a plaque with a fibrous cap measuring <65 *μ*m. OCT-detected TCFAs are often observed in the culprit lesions of acute coronary syndrome.

**Figure 5:**
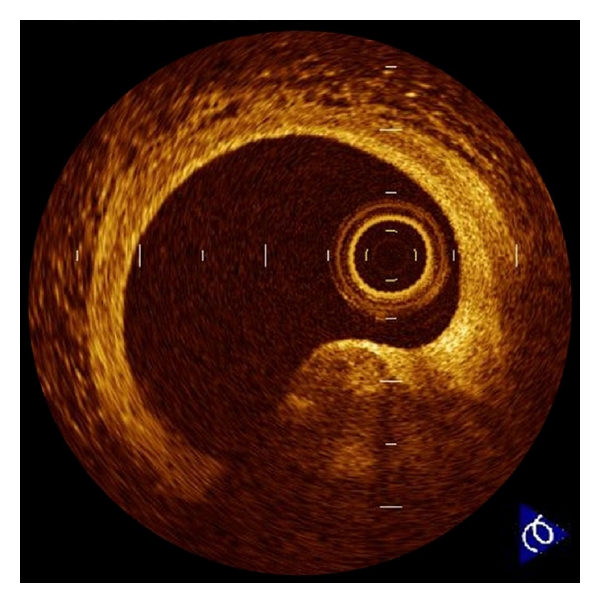
Calcified nodule. Calcified nodule is defined as a protrusion of a signal-poor or heterogeneous region with a sharply delineated border. The origin of this lesion is not precisely known, but it appears to be associated with healed plaques.

Optical coherence tomography (OCT) is a recently developed intravascular imaging modality using near-infrared light to create images [1–3]. The greatest advantage of OCT is its high resolution (10 to 20 *μ*m), which is 10 times higher than that of intravascular ultrasound (IVUS). OCT can discriminate three layers of the coronary artery wall demonstrating the intima as the signal rich layer nearest the lumen, the media as the signal poor middle layer, and the adventitia as the signal rich layer surrounding the signal poor layer of the media [4]. With regard to tissue characterization, OCT allows us to identify three types of coronary plaques, such as fibrous, fibrocalcific, and lipid. Fibrous plaque is characterized by signal rich, homogenous lesion, fibrocalcific plaque by signal poor, sharp border lesion, and lipid rich plaques as signal poor, diffuse border lesion [5]. A histology-controlled OCT study showed good intra- and interobserver reliabilities (*κ* = 0.83-0.84) and high sensitivity and specificity in each plaque demonstrating 71–79% and 97-98% for fibrous plaques, 95-96% and 97% for fibrocalcific plaques, and 90–94% and 90–92% for lipid-rich plaques, respectively [5, 6]. Furthermore, OCT can detect plaque rupture (Figure 1), erosion (Figure 2), intracoronary thrombus (Figure 3), thin-cap fibroatheroma (TCFA; Figure 4), and calcified nodule (Figure 5) [7–9]. Although there are some limitations including shallow penetration depth of the infra-red light and complex procedure of image acquisition, the high resolution of OCT provides more detailed structural information of the coronary artery wall compared with conventional imaging modalities [10, 11]. Thus, OCT has been applied for the assessment of culprit lesion morphologies in patients with acute coronary syndrome (ACS).

## 2. Pathology of Culprit Lesions in ACS

Autopsy studies have demonstrated that ACS results from sudden luminal narrowing caused by thrombosis based on plaque rupture, erosion, and superficial calcified nodule [12, 13]. In these coronary features, plaque rupture is the most frequent (55 to 60%), plaque erosion to be the second (30 to 35%), and superficial calcified nodule to be the least (2 to 7%) [12]. Plaque rupture is identified by a presence of fibrous cap discontinuity and a cavity formation within the plaque. Atheroma with thin-fibrous cap of <65 *μ*m is thought to be precursor lesion of plaque rupture. Erosion has an area lacking surface endothelium and occurs over lesion with thick intima. Calcified nodule is a plaque with luminal thrombi showing calcific nodule protruding into the lumen through a disrupted thin fibrous cap. Based on these pathohistological findings, thrombosis-prone vulnerable plaques are characterized by 5 major and 5 minor features as listed in Table 1 [13]. Compared with conventional imaging modalities, OCT has an ability to identify these features more precisely in vivo.

## 3. OCT Assessment of Culprit Lesions in ACS

The first OCT study to assess in vivo culprit lesion morphology in patients with ACS was conducted by Jang et al. [14]. They used a 3.2 Fr. proto-type OCT catheter and revealed higher frequency of TCFA in ACS compared with stable angina pectoris (SAP) (72% in acute myocardial infarction (AMI), 50% in unstable angina pectoris (UAP), and 20% in SAP; *P* = 0.012). However, this study showed lower frequency of thrombus and plaque rupture in AMI in comparison with previous pathological reports [12, 13]. The thick OCT catheter, the time delay between the symptom onset and imaging (4.6 ± 5.3 days), and the thrombolysis and/or antiplatelet therapy before the imaging might affect the results. Thereafter, Kubo et al. [10] used commercially available OCT system with a 0.014 inch optic fiber, IVUS, and angioscopy in AMI within 6 hours from symptom onset. This study showed superiorities of OCT for the detection of plaque rupture (73% versus 40% versus 43%, *P* = 0.021), erosion (23% versus 0% versus 3%, *P* = 0.003), and thrombus (100% versus 33% versus 100%, *P* < 0.001) compared with IVUS and angioscopy. The frequency of OCT-detected plaque rupture, erosion, and thrombus was similar to that of the pathological reports. The frequency of OCT-detected TCFA was 83%, and the thickness of fibrous cap was 49 ± 21 *μ*m in AMI. Tanaka et al. [15] used OCT to compare the ruptured fibrous cap thickness and the rupture site between exertion-triggered and rest-onset ACS. In the results, rest-onset ACS had thinner ruptured fibrous-cap thickness (50 *μ*m versus 90 *μ*m, *P* < 0.010) and more frequent rupture near the shoulder of the plaque (57% versus 93%, *P* = 0.014) in comparison with exertion-triggered ACS. Interestingly, not only TCFAs but also thick cap (up to 150 *μ*m) fibroatheromas were ruptured in exertion-triggered ACS, and the high-sensitive C-reactive protein level was negatively correlated with the thickness of the ruptured fibrous-cap. Exercise-induced high shear stress at the site of the plaque shoulder and fibrous-cap inflammation might be associated with the fibrous-cap disruption of >65 *μ*m thick. By using OCT, Ino et al. [16] showed differences of ruptured plaque morphologies between ST-segment elevated AMI and non-ST-segment elevated ACS. Although the minimum lumen area was similar in both groups, the ruptured cavity size was significantly larger in ST-segment elevated AMI compared with non-ST-segment elevated ACS. Furthermore, the ruptured plaque of which aperture was open wide against the direction of coronary flow was more often seen in ST-segment elevated AMI compared with non-ST-segment elevated ACS (46% versus 17%, *P* = 0.036). The morphological feature of plaque rupture could relate to the clinical presentation in patients with ACS. Mizukoshi et al. [17] reported that the frequency of plaque rupture (43% versus 13% versus 71%, *P* < 0.001) and plaque erosion (32% versus 7% versus 8%, *P* = 0.003) was significantly different among the types of UAP; Braunwald class I, II, and III. The fibrous cap thickness (140 *μ*m versus 150 *μ*m versus 60 *μ*m, *P* < 0.001), minimal lumen area (0.70 mm^2^ versus 1.80 mm^2^ versus 2.31 mm^2^, *P* < 0.001), and the frequency of thrombus (72% versus 30% versus 73%, *P* < 0.001) were also different among the types of UAP. This clinical OCT study disclosed that the culprit plaque in class III UAP might be more vulnerable than the other classes. 

Unstable lesion morphologies before percutaneous coronary intervention (PCI) affect the outcome after the procedure. An observational OCT study [18] demonstrated that TCFA was often seen at target lesions of the patients with noreflow after PCI compared with good reflow (50% versus 16%, *P* = 0.005). The frequency of the noreflow phenomenon increased according to the size of the lipid arc as determined by OCT. A serial OCT study [19] showed markedly different vascular response up to 9 months after drug-eluting stent implantation between the patients with UAP and SAP. Acute stent malapposition (67% versus 32%, *P* = 0.038) and tissue protrusion after PCI (79% versus 42%, *P* = 0.005) were observed more frequently in the UAP patients. Plaque rupture was significantly increased after PCI in the UAP patients (42% to 75%, *P* = 0.018), and the persistence of core cavity after plaque rupture at 9-month' followup (28% versus 4%, *P* = 0.031) was observed more frequently in the UAP patients compared with the SAP patients. At 9 months' follow-up, the frequency of malapposed stent (33% versus 4%, *P* = 0.012) and partially uncovered stent by neointima (72% versus 37%, *P* = 0.019) was significantly greater in the UAP patients than that in the SAP patients. 

**Figure 6:**
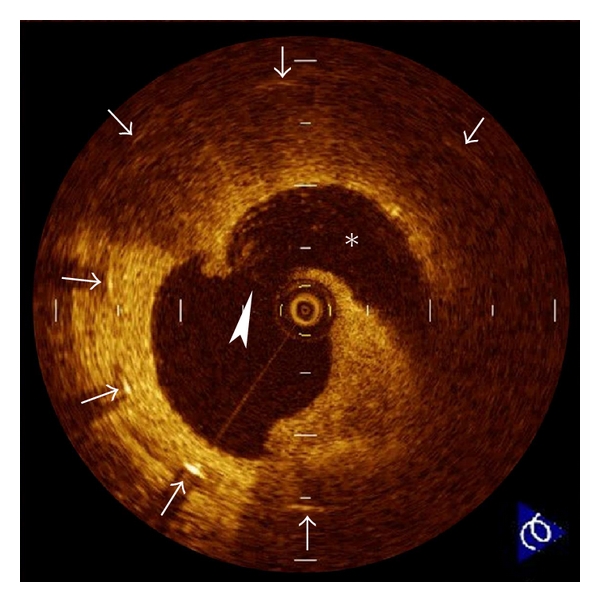
Neointimal plaque rupture. A 71-year-old male was admitted to our hospital with very late (>10 years) bare-metal stent thrombosis. OCT disclosed a plaque rupture (arrowhead) and cavity formation (∗) within neointima in the stented segment (arrows).

Recent OCT studies have suggested the development of in-stent neoatherosclerosis after PCI. Kashiwagi et al. [20] and Nishiguchi et al. [21] investigated the lesions with very late stent thrombosis by using OCT and demonstrated lipidic plaque formation, TCFA, and plaque rupture within the neointima in the stented segments. Although late stent thrombosis is thought to be associated with delayed endothelialization, these reports highlight that it can occur despite full stent coverage. Atherosclerotic developments within the neointima might play an important role in very late stent thrombosis (Figure 6).

## 4. OCT-Detected Vulnerable Plaques

The term “vulnerable plaque” is used to describe thrombosis-prone plaques. The precursor lesion for plaque rupture is characterized by a thin fibrous cap heavily infiltrated macrophages and an underlying lipid core. Virmani et al. [12] defined plaque vulnerability based on the actual thickness of the histological section from measurements made of plaque ruptures. TCFA was defined as a lesion with a fibrous cap <65 *μ*m thick. A thickness of 65 *μ*m was chosen as a criterion of instability because in rupture the mean cap thickness was 23 ± 19 *μ*m; 95% of caps measured less than 65 *μ*m within a limit of only two standard deviations. At present, OCT might be the best tool to detect TCFA in vivo [22–25]. Kume et al. [7] examined 35 lipid-rich plaques from 38 human cadavers and demonstrated a good correlation of the fibrous cap thickness between OCT and histology (*r* = 0.90; *P* < 0.001). In the clinical setting, Fujii et al. [26] showed that OCT-detected TCFA was associated with high-sensitive C-reactive protein, and its distribution in the coronary artery tree was similar to that in the previous autopsy reports. Kashiwagi et al. [27] used multidetector computed tomography to compare lesion characteristics between OCT-detected TCFA and non-TCFA. Positive remodeling (76% versus 31%, *P* < 0.001) and ring-like enhancement (44% versus 4%, *P* < 0.001) as determined by multidetector computed tomography were observed more frequently in OCT-detected TCFAs than in non-TCFAs. Computed tomography attenuation value was significantly lower in OCT-detected TCFAs than that in the non-TCFAs (35.1 ± 32.3 HU versus 62.0 ± 33.6 HU, *P* < 0.001). Kubo et al. [28] assessed the relationship between plaque color evaluated by coronary angioscopy and fibrous cap thickness estimated by OCT in vivo. As a result, there was a significant negative correlation between yellow color intensity and fibrous cap thickness (*P* < 0.001). Furthermore, 80% of intensive yellow plaques were OCT-detected TCFAs with a cap thickness of <65 *μ*m. Sawada et al. [29] compared the feasibility for detecting TCFA between OCT and virtual histology IVUS. Although the positive ratio of virtual histology IVUS for detecting TCFA was 45.9%, that of OCT was 77.8%. On top of its reliability as a tool to measure the fibrous-cap thickness in vivo, a recent OCT study conducted by Takarada et al. [30, 31] demonstrated that the lipid-lowering therapy with statin for 9 months significantly increased the fibrous-cap thickness in patients with hypercholesterolemia (151 ± 110 to 280 ± 120 *μ*m, *P* < 0.001). As therapies to prevent or make regression of atherosclerosis are developed, OCT can help to assess the treatment efficacy for plaque stabilization.

**Figure 7:**
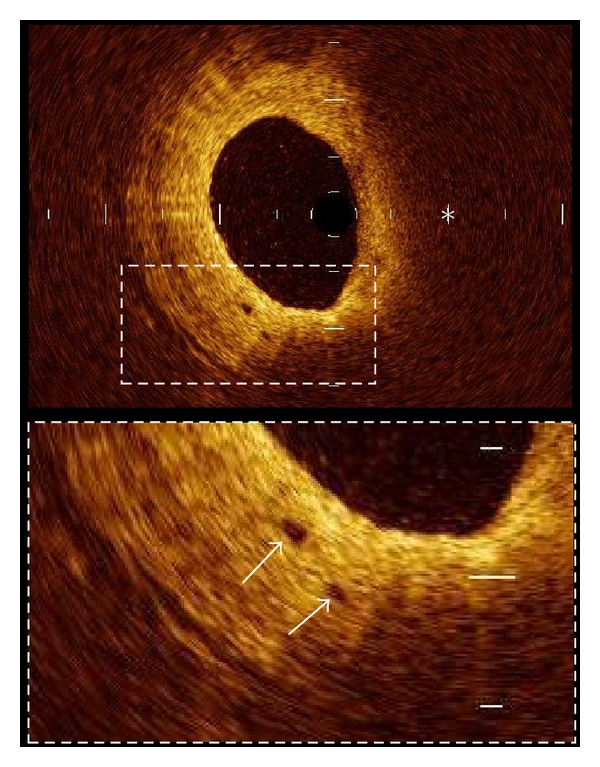
Plaque neovascularization. Microvessels within the intima (arrows) appear as signal poor voids that are sharply delineated. Two microvessels are located in thickened intima at 7 o'clock position. (∗ = lipid).

Plaque neovascularization is a common feature of vulnerable plaque. Proliferation of microvessels is considered to be related to intraplaque hemorrhage and plaque destabilization. The high resolution of OCT provides an opportunity to detect plaque neovascularization in vivo (Figure 7). Kitabata et al. [32] demonstrated increase of microvessels density in OCT-detected TCFAs in vivo. The presence of microvessels in the plaques was also associated with positive vessel remodeling and elevated high-sensitive C-reactive protein levels. The OCT evaluation of microvessels density might be helpful to assess plaque vulnerability.

**Figure 8:**
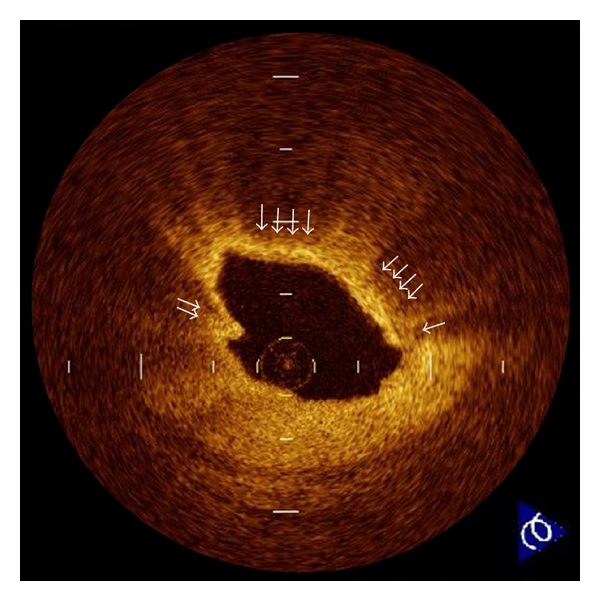
Macrophages. Macrophages (arrows) are seen as signal-rich, distinct, or confluent punctuate regions that exceed the intensity of background speckle noise. The high contrast and resolution of OCT enable the quantification of macrophages within fibrous caps of atherosclerotic plaques.

A unique aspect of OCT is its ability to visualize the macrophages (Figure 8). Tearney et al. [33] and MacNeill et al. [34] descried the potential of OCT to estimate macrophage accumulation within fibrous caps. There was a high degree of positive correlation between OCT and histological measurements of fibrous cap macrophage density (*r* < 0.84, *P* < 0.0001). A range of OCT signal standard deviation thresholds (6.15% to 6.35%) yielded 100% sensitivity and specificity for identifying caps containing >10% CD68 staining in their study. 

## 5. Limitations of OCT

The current commercially available time-domain OCT system requires vessel occlusion by means of gentle balloon inflation and vessel flushing with lactated Ringers' solution or saline infusion at the time of image acquisition because the near-infrared light signals are attenuated by red blood cells. Therefore, the assessment of long coronary segment and the observation of left main coronary artery might be limited. Inadequate displacement of blood can be a problem in vessels >3.5 mm in diameter, where large bifurcations are present and in the presence of competitive flow from collaterals or bypass grafts. In addition, there is concern about the local consequences of balloon inflation. To overcome this limitation, a simplified technique for coronary blood removal, which was achieved through continuous injection of contrast agents or dextran with lactate Ringers' solution, is recommended [35, 36]. This nonocclusive technique of OCT image acquisition is safe and useful and promises to reduce the procedural time. A further limitation of OCT is the relatively shallow axial penetration depth of 2 mm. The OCT signal does not reach the back wall of thick atherosclerotic lesions. The penetration depth of OCT depends on tissue characteristics. Lipid-rich plaque or coronary thrombus causes OCT signal attenuation, which interrupts to observe deep layers of coronary artery wall. OCT is not appropriate for the quantification of lipid-core size and the evaluation of arterial remodeling. This drawback may affect the role of OCT for assessment of lesion instability. The current OCT is well suited for the assessment of the plaque morphologies within 500 *μ*m of the luminal surface.

## 6. Future Perspectives of OCT

Recently, a second-generation OCT technology, named frequency-domain OCT, has been developed. This new technology will solve the current time-domain OCT limitations by imaging at much higher frame rates with slightly deeper penetration depth and greater scan area [37–39]. In combination with a short, nonocclusive flush and rapid spiral pullback, the higher frame rates generated by frequency-domain OCT enable imaging of the 3-dimensional microstructure of longer segments of coronary arteries [40]. In addition, frequency-domain OCT facilitates the acquisition of spectroscopic and polarization data for tissue characterization [41]. The development of frequency-domain OCT would allow easy and precise identification of vulnerable plaque in daily clinical practice.

## 7. Conclusions

The high resolution of OCT provides histology-grade definition of the microstructures of coronary unstable plaques in vivo. OCT can visualize unstable lesion morphologies in vivo which have been demonstrated by histological examinations. Thus, OCT allows a greater understanding of the pathophysiology of ACS and may have a potential to propose guidance for the appropriate patient-specific therapeutic approach. Although more clinical research with greater number of subjects and development of the imaging technology are required, OCT will play an important role in the future cardiology.

## Figures and Tables

**Table 1:** Criteria for defining vulnerable plaques [13].

Major criteria
(1) Active inflammation (monocyte/macrophage and sometimes
T-cell infiltration)
(2) Thin cap with large lipid core
(3) Endothelial denudation with superficial platelet aggregation
(4) Fissured plaque
(5) Stenosis > 90%
Minor criteria
(1) Superficial calcified nodule
(2) Glistening yellow
(3) Intraplaque hemorrhage
(4) Endothelial dysfunction
(5) Outward (positive) remodeling

## Abbreviations and Acronyms

ACS: Acute coronary syndrome
AMI: Acute myocardial infarction
IVUS: Intravascular ultrasound
OCT: Optical coherence tomography
PCI: Percutaneous coronary intervention
SAP: Stable angina pectoris
TCFA: Thin cap fibroatheroma
UAP: Unstable angina pectoris.
